# Evaluation of *Staphylococcus Aureus* and *Pseudomonas Aeruginosa* in Saliva of Patients with Acute Myeloid Leukemia

**DOI:** 10.30476/dentjods.2023.97098.1989

**Published:** 2024-03-01

**Authors:** Jannan Ghapanchi, Hanieh Farahmand, Abdollah Bazargani, Seyed Omid Reza Zekavat, Fatemeh Lavaee, Amir Hossein Ojaghi

**Affiliations:** 1 Oral and Dental Diseases Research Center, Dept. of Oral Medicine, School of Dentistry, Shiraz University of Medical Sciences, Shiraz, Iran; 2 Post Graduate Student, Dept. of Oral and Maxillofacial Medicine, School of Dentistry, Shiraz University of Medical Sciences, Shiraz, Iran; 3 Dept. of Medicine of Microbiology, School of Medicine, Shiraz University of Medical Sciences, Shiraz, Iran; 4 Dep.t of Pediatric, Division of Pediatric Hematology and oncology, School of Medicine, Shiraz University of Medical Sciences, Shiraz, Iran; 5 Dental Research Committee, School of Dentistry, Shiraz University of Medical Sciences, Shiraz, Iran

**Keywords:** *Staphylococcus aureus*, *Pseudomonas aeruginosa*, Saliva, Acute myeloid leukemia

## Abstract

**​Statement of the Problem:**

Patients with leukemia are prone to infectious and often life-threatening complications. Evidence suggests that a specific oral microbiota may contribute to septicemia, which can delay antineoplastic treatment, compromise treatment efficacy, or even endanger patients' lives.

**Purpose:**

This study investigated the prevalence of *Staphylococcus aureus* and *Pseudomonas aeruginosa* in the saliva of patients with acute myeloid leukemia who were candidates for bone marrow transplantation.

**Materials and Method:**

This cross-sectional study was conducted in 2019 in the Hematology-Oncology Department of Namazi Hospital, Shiraz University of Medical Sciences. The study included 28 patients with acute myeloid leukemia eligible for bone marrow transplantation as the case group and age- and sex-matched healthy individuals as the control group. Unstimulated saliva samples were collected to determine the frequency of *Staphylococcus aureus* and *Pseudomonas aeruginosa*. Data were analyzed using SPSS version 18, the chi-square test, and the independent t-test.

**Results:**

In the patients with acute myeloid leukemia, 26 (86%) were positive for *Staph-ylococcus aureus* and 18 (60%) were positive for *Pseudomonas aeruginosa*. In the healthy group, 11 (22.9%) were positive for *Staphylococcus aureus* and 3 (6.2%) were positive for *Pseudomonas aeruginosa*. The frequency of *Pseudomonas aeruginosa* and *Staphylococcus aureus* in the saliva samples of patients with acute myeloid leukemia was significantly higher than in the healthy control subjects (*p* value < 0.05). Chi-square test showed no significant association between age and the frequency of bacteria (*p* value= 0.27).

**Conclusion:**

In the current study, the frequency of *Staphylococcus aureus* and *Pseudomonas aeruginosa* in the saliva of patients with acute myeloid leukemia was higher than in the healthy control group.

## Introduction

Malignant neoplasms, including hematopoietic dyscrasias, are an important cause of death in many countries [ 1 ]. Acute leukemia is a clonal hematopoietic stem cell dyscrasia of malignant cells with uncontrolled growth or failure of differentiation, classified according to myeloid or lymphoid origin. In acute myeloid leukemia (AML), there is an accumulation of poorly differentiated and nonfunctional myeloblasts, the specific cause of which is usually unknown in various patients [ 2 ].

Patients with leukemia are typically prone to infectious or hemorrhagic and often life-threatening complications [ 3 ]. The oral cavity contains several species of microflora that are harmless in healthy individuals. Since some of these flora species are opportunistic, an impaired immune response in cancer patients provides the prerequisite for them to become pathogens. The immune response is disturbed by the patients neoplasm and the treatments prescribed to them, including chemotherapy, radiation therapy, and bone marrow transplantation. The disturbed homeostasis may affect the integrity of the oral mucosa, providing a suitable condition for opportunistic infections and their spread, leading to a life-threatening condition [ 4 ]. The oral cavity is a reservoir for pathogenic organisms that can cause microbial spread to distant body sites in immunocompromised hosts [ 5 ]. *Staphylococcus aureus* is a facultative aerobic, gram-positive bacterium responsible for skin, bone, and joint infections, medical implants, and food poisoning [ 6 ]. *Pseudomonas aeruginosa* is a rod-shaped, encapsulated, facultative aerobic, gram-negative bacterium [ 7 ]. This species is of great medical importance [ 8 ] because it is opportunistic for healthy individuals and a pathogen for immunocompromised patients [ 9 ]. *Pseudomonas aeruginosa* promotes the highest mortality rate in patients with neutropenia and those have received bone marrow transplantation [ 10 ]. In addition, *Pseudomonas aeruginosa* is frequently isolated in post-transplant pneumonia [ 11 ]. *Staphylococcus aureus* and *Pseudomonas aeruginosa* are two dangerous opportunistic bacteria that can lead to mucositis and hospital-acquired infections [ 12 ].

Studies have shown that the oral microbial flora changes qualitatively in cancer patients [ 13 ]. Higher prevalence of cariogenic oral microflora, including *Streptococcus mutans* and *Lactobacillus*, as well as higher plaque index and severe oral inflammation in patients undergoing chemotherapy has been reported [ 14 ]. *Staphylococci* have been introduced as a cause of systemic infections in children with malignancies [ 15 ]. In studies, different bacterial species were isolated from lymphoma patients who had received chemotherapy. This study confirmed that hospitalized patients with leukemia and lymphoma might be frequently infected with *Pseudomonas aeruginosa* [ 16 ].

While there are many reports of the increased prevalence and pathogenicity of the oral microbiota and the increasing risk of antibiotic resistance, the local epidemiologic studies of bacterial infections in immunocompromised patients, including AML, may influence the approach to antibiotic treatment, prophylaxis, and infection management. Inadequate infection control and its side effects may compromise the efficacy of cancer treatment and delay the antineoplastic protocol [ 17 ].

Considering the pathogenicity and multidrug resistance of *Staphylococcus aureus* and *Pseudomonas aeruginosa*, this study was conducted to determine the frequency of these bacteria in the saliva of patients with acute AML who are candidates for bone marrow transplantation.

## Materials and Method

This cross-sectional study was conducted in 2019 in the Hematology-Oncology Department of Namazi Hospital, Shiraz University of Medical Sciences. The study protocol was approved by the Ethics Committee of Shiraz University of Medical Sciences, Shiraz, Iran (code IR.SUMS. DENTAL.REC.1398.122), and was designed in accordance with the *Helsinki Declaration* [ 18 ]. Twenty-eight patients with AML who were eligible for bone marrow transplantation and hospitalized in the Hematology-Oncology Department of Namazi Hospital, Shiraz University of Medical Sciences, participated in this study (Group 1). In addition, 48 healthy control subjects (Group 2) were selected from individuals who had been referred to Shiraz Dental School. Participants with severe periodontal disease (clinical attachment loss of more than 5 mm) [ 19 ], smokers and patients under radiotherapy or chemotherapy were excluded from the study. AML patients with other immunosuppressive and autoimmune diseases and patients under 18 years of age were also excluded. In the case and control groups, teeth were brushed at least once a day. Participants with autoimmune diseases or immunosuppressive conditions were excluded from the healthy control group. Patients in both the case and control groups had not used antimicrobials or mouth rinses for at least two weeks prior to saliva collection. The study protocol was explained to both groups, and participants provided written informed consents.

Participants were not allowed to eat or drink for 1 hour before sample collection. Saliva samples were collected from 28 patients with AML who were eligible for bone marrow transplantation as the case group and from 48 age-matched healthy volunteers as the control group. Saliva samples were collected for detection of *Staphylococcus aureus* and *Pseudomonas aeruginosa*.

Spitting is a method of saliva collection in which saliva is collected from the floor of the mouth and then spit into a container equipped with a measuring device, such as a funnel linked to a tube/container. This method minimizes the evaporation of saliva in long-term samples and it can be employed when saliva flow is very low; though, it might have stimulatory effects. Subjects were instructed to spit saliva into sterile Falcon tubes containing 3 ml of thioglycollate broth [ 20 ]. Saliva samples were immediately sent to the microbiology laboratory under cold chain conditions and cultured on eosin methylene blue agar (EMB) to select gram-negative bacteria, which were incubated at 37°C for 24 to 48 hours. Bacteria colonies (different colonies per sample) were centrifuged at 17000 g (12500 rpm) for 10 minutes. The supernatant was discarded, and the deposit was resuspended in 1 ml of phosphate-buffered saline to obtain a concentrated suspension. Using the standard strip plate method, a loop full of concentrated suspension was inoculated onto EMB, McConkey's agar culture medium (Germany). All culture plates were incubated at 37°C for 24 hours, and bacterial growth was observed as pink and white colored colonies. Colonies were primarily identified using the API 20E system according to the manufacturer's protocol (BioMerieux® SA, France). The API20E strip was used for the identification of gram-negative rods. All glycerol samples were aliquoted without dilution and stored at -80°C until molecular assays were performed. The presence of *Staphylococcus aureus* and *Pseudomonas aeruginosa* was examined. All data were analyzed using SPSS version 18; the chi-square test was used to compare the abundance of these two bacteria. An independent t-test was also used to compare the mean age of the two groups.

## Results

In this study, 48 saliva samples from healthy controls and 28 patients with AML who were candidates for bone marrow transplantation were examined. The mean age of the healthy controls and patients with AML was 33.56±12.45 years, and that of the cases was 30.36±17 years. The independent-samples test showed no significant difference between the ages of the two groups (*p* value= 0.35). The sex distribution is shown in Table 1. The frequency of *Staphylococcus aureus* and *Pseudomonas aeruginosa* is shown in Table 2.

**Table 1:** Sex distribution in both evaluated groups

Groups (N)	Sex	Frequency	Present
Healthy Control (48)	Female	30	62.5%
	Male	18	37.5%
Patients with AML (28)	Female	14	50%
	Male	14	50%

**Table 2:** The frequency of *Staphylococcus aureus* and *Pseudomonas aeruginosa* in both evaluated groups

	*Staphylococcus aureus*		*Pseudomonas aeruginosa*	
	Positive	Negative	Positive	Negative
Case	26	2	18	10
Control	11	37	3	45
*p* value	0.001		0.0001	

The frequency of *Pseudomonas aeruginosa* in the saliva samples of AML patients was significantly higher than in the healthy controls. The chi-square test showed a significant difference between bacterial infections in both groups (*p* value < 0.05). Chi-square test revealed no significant association between age and bacterial frequency (*p* value=0.27).

## Discussion

In the current study, the frequency of *Staphylococcus aureus* and *Pseudomonas aeruginosa* was higher in AML than in the healthy control group (*p*< 0.05), consistent with the results of previous studies. A mini-review reported that despite fluoroquinolone prophylaxis in neutropenic patients, bacteremic episodes caused by *Staphylococcus aureus* and *Pseudomonas aeruginosa* occurred in patients receiving HCT [ 22 ]. Al-kuhla *et al*. [ 23 ] isolated *Staphylococcus aureus* from the saliva of lymphoma patients receiving chemotherapy. In debilitated patients, the oral cavity can be a reservoir for *Pseudomonas aeruginosa*. Eduardo *et al*. [ 24 ] reported that antibiotic-resistant oral ulcers contribute to *Pseudomonas aeruginosa* in these patients. *Staphylococcus aureus* causes bacteremia in 9-10% of adults with malignancies, with a mortality rate of 15-24%; the mortality rate for *Staphylococcus aureus* pneumonia is approximately 49.5% in cancer patients [ 25 ]. A systematic review evaluated the oral microbiota of patients undergoing chemotherapy for cancer in 17 studies, listing the most common bacteria. *Pseudomonas spp.* and *Staphylococcus spp*. were the most frequently observed bacteria in this list, along with several other spices [ 26 ]. The same study was performed on 13 clinical trials with 300 patients suffering from different types of cancer, and *Pseudomonas spp.* and *Staphylococcus spp.* were in the list of the most frequently isolated gram-negative and gram-positive species, respectively [ 27 ].

*Staphylococcus aureus*, *Pseudomonas aeruginosa*, and some other pathogens were more frequently isolated from the saliva of patients with oral cancer [ 27 ].

In another study, a significant increase in *Candida spp.* and coagulase-negative Staphylococci was observed during the treatment phase of immunocompromised patients with hematologic malignancies, while *Streptococci* showed no change compared to other treatment phases [ 28 ]. Al-kuhla *et al*. [ 23 ] observed a higher prevalence of staphylococci during chemotherapy in lymphoma patients. Worldwide, many patients undergo hematopoietic cell transplantation (HCT) [ 29 ]. In recent years, the need for improved oral hygiene in cancer patients is confirmed [ 30 ]. In the past, the treatment of transplant cases was not part of dental education. Evidence has confirmed the correlation between oral microflora and systemic diseases [ 31 ]. It is recommended to inform the patients about the adverse effects of HCT on the oral mucosa prior conditioning. This encourages them to consider oral prophylaxis and to pay careful attention to oral hygiene. According to reports in the literature, only 30% of bone marrow transplant recipients are educated about oral hygiene prior to their transplant [ 32 ]. Bone marrow transplantation is one of the treatment options for AML patients. Since AML patients are immunosuppressed, they are more susceptible to bacterial and fungal infections, severe bacteremia, and various systemic dangerous and life-threatening infections, which can be treated by eliminating the microbial load. The recommended protocols for all bone marrow candidates are oral hygiene education, dental examination, and treatment prior to initiation of therapy. Immunocompromised patients, including HCT candidates, should improve their oral hygiene with regular rinsing with normal saline or sodium bicarbonate solution, tooth brushing, and atraumatic flossing. Microbial screening in hospitalized patients with hematopoietic malignancies at various stages of treatment is recommended [ 33 ].

Patient recruitment in our study was hampered by limited funding. Patient cooperation was low.

A larger sample size is recommended to obtain more accurate assessments. Because the emphasis on hygiene recommendations in treatment protocols for immunocompromised patients or patients eligible for bone marrow transplantation is not the same in different countries, this may be an important issue for future evaluations. In addition, access to other oral pathogens in patients with other types of malignancies is suggested.

## Conclusion

The incidence of *Staphylococcus aureus* and *Pseudomonas aeruginosa* was significantly higher in AML patients who were candidates for bone marrow transplantation than in healthy participants. This finding confirms the importance of improved oral hygiene in patients with leukemia.
